# Advances of target therapy on NOTCH1 signaling pathway in T-cell acute lymphoblastic leukemia

**DOI:** 10.1186/s40164-020-00187-x

**Published:** 2020-11-13

**Authors:** Ruyue Zheng, Menglin Li, Shujuan Wang, Yanfang Liu

**Affiliations:** Department of Hematology, The First Affiliated Hospital of Zhengzhou University, Zhengzhou, 450052 China

**Keywords:** T-cell acute lymphoblastic leukemia, *NOTCH1*, γ-Secretase inhibitors, SERCA inhibitors, Monoclonal antibody

## Abstract

T-cell acute lymphoblastic leukemia (T-ALL) is one of the hematological malignancies. With the applications of chemotherapy regimens and allogeneic hematopoietic stem cell transplantation, the cure rate of T-ALL has been significantly improved. However, patients with relapsed and refractory T-ALL still lack effective treatment options. Gene mutations play an important role in T-ALL. The *NOTCH1* gene mutation is the important one among these genetic mutations. Since the mutation of *NOTCH1* gene is considered as a driving oncogene in T-ALL, targeting the *NOTCH1* signaling patheway may be an effective option to overcome relapsed and refractory T-ALL. This review mainly summarizes the recent research advances of targeting on *NOTCH1* signaling pathway in T-ALL.

## Background

T-cell acute lymphoblastic leukemia (T-ALL) is a hematological malignancy originating from T-lymphocytes in bone marrow. In recent years, with the advances of chemotherapy and the application of allogeneic hematopoietic stem cell transplantation in the management of T-ALL, the outcomes of this disease have been significantly improved. At the same time, the use of chimeric antigen receptor modified T cell (CAR-T) therapy also provides new and effective immunotherapy for T-ALL [1]. However, some T-ALL patients are refractory to induction therapy, and others responding to induction therapy may relapse and become refractory to salvage therapy. There are no effective treatment options for those patients with poor prognosis. Thus the underlying molecular mechanisms and therapy for refractory and relapsed T-ALL are the main focuses of current researches. With the advance of state-of-art molecular technology such as next generation DNA sequencing, studies have found that germline or somatic mutations of some genes may play important roles in the occurrence, development, and drug-resistance of T-ALL [2]. The mutation in *NOTCH1* gene is one of the important genetic mutations in T-ALL [3, 4]. Studies have shown that *NOTCH1* mutations play a role in carcinogenesis or tumor suppression under different cell backgrounds [5, 6]. In T-ALL, *NOTCH1* is a driving oncogene, and the dominant active mutations induce the development of pre-T cells to leukemia [7–9]. Therefore, further understandings of the *NOTCH1* mutation in T-ALL will shed light on developing targeting therapy for T-ALL patients. Targeting the *NOTCH1* signaling pathway may be an optimal management for the treatment of relapsed and refractory T-ALL carrying *NOTCH1* mutation.

## NOTCH1 gene and the signaling pathways

*NOTCH1* gene is a member of a highly conserved *NOTCH* gene family (*NOTCH1-4*), located on chromosome 9q34.3, and encodes a NOTCH1 transmembrane signal protein. The NOTCH1 receptor protein is composed of three regions/subunits: (1) The extracellular region consisting of the epidermal growth factor (EGF)-like repeats, the negative regulatory region (NRR) composed of 3 cysteine-rich Lin12-Notch repeats (LNR) and a heterodimerization domain (HD); (2) The transmembrane region that includes the site of action for ADAM protease and γ-secretase; (3) The intracellular region consisting of a proline/glutamic acid/serine/threonine enriched motif (PEST) domain, which is primarily responsible for producing NOTCH1 active component ICN1 [10–12].

The mature NOTCH1 receptor protein containing the heterodimerization domain is transported to the cell surface. The EGF-like repeats in the extracellular region combine with NOTCH ligands (Jag1, Jag2, Dll1, Dll3, etc.) in neighboring cells, leading to the exposure of S2 site and S3 site in NRR region near the cell membrane. As a result, this conformation change triggers the proteolytic function of the ADAM protease in S2 site and the γ-secretase in S3 site, leading to the release of the intracellular region ICN1. ICN1 enters the nucleus and interacts with the DNA-binding protein continuous spontaneous localization (CSL) and the Mastermind-like (MAML) family proteins, resulting in the formation of a transient ICN1-CSL-MAML complex and activation of downstream gene transcription. Finally, the PEST region binds to the FBXW7 that includes E3 ubiquitin ligase. The NOTCH-FBXW7 complex recognizes and degrades ICN1 to terminate the signaling pathway [12–14].

## Agents target on NOTCH1 pathway

**Fig. 1:**
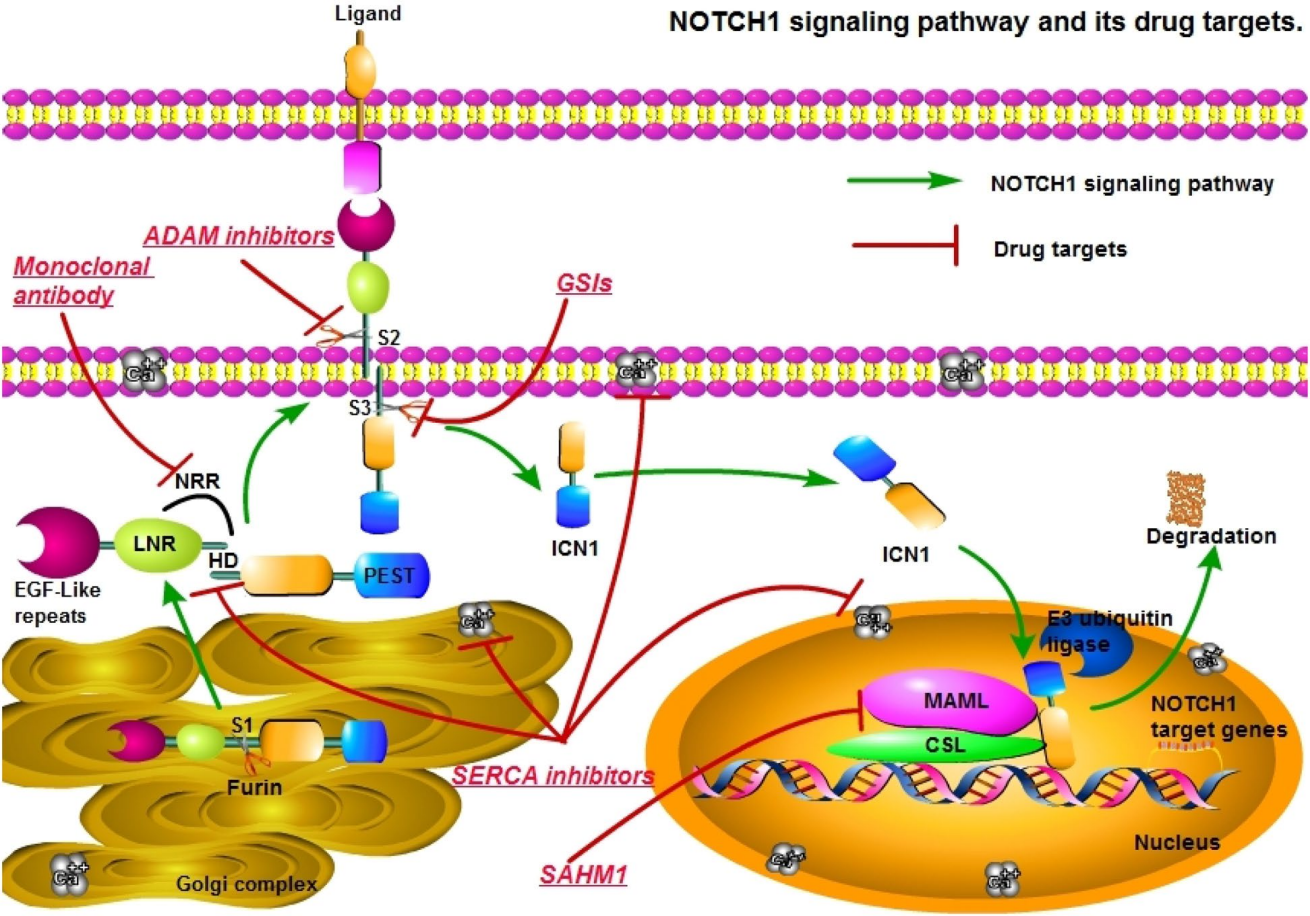
NOTCH1 signaling pathway and drug targets. Agent categories and their targets: ADAM inhibitors prevent ADAM protease S2 site digestion; γ-secretase inhibitors (GSIs) prevent the cleavage of S3 site of γ-secretase; Monoclonal antibody prevent the conformational change of the negative regulatory region (NRR) from exposing the S2 and S3 cleavage sites; SERCA inhibitors include Ca^2+^ ATPase inhibitors prevent transportation process of NOTCH1 and the formation of heterodimerization domain (HD); SAHM1 prevent the formation of ICN1-CSL-MAML complex

There have been many small molecules tested in the targeted therapy on *NOTCH1* pathway in T-ALL [15, 16], which can be divided into the following categories (Fig. 1).

### γ-Secretase inhibitors (GSIs)

γ-Secretase is a key enzyme in the activation process of NOTCH pathway. Inhibiting its activity can induce G0/G1 cell cycle arrest and inhibit cell proliferation.

The original GSI trial drug was MK-0752. Seven patients with T-ALL received MK-0752 for 56 days. One of the patients showed a certain anti-leukemia effect to the drug, but the response was transient. Most patients developed gastrointestinal toxicity (mainly diarrhea) at a dose of 300 mg/m^2^, which lead to the termination of the trial [17]. The GSI may block NOTCH1 and NOTCH2 at the same time and disturbs the normal function of physiological NOTCH signals. As a result, serious imbalance of digestive tract homeostasis is considered as the main reason of diarrhea [2, 18].

PF-03084014 is an oral, non-competitive, reversible, selective γ-secretase inhibitor. In a HPB-ALL (a subset of human T-ALL) cell line, cells in S-G2-M phase were suppressed after 7 days of PF-03084014 treatment, while cells in G0-G1 phase were accumulated. The ICN1 protein was completely inhibited, and the expression of *Notch* target genes *Hes-1* and *cMyc* were down-regulated in these cells [19]. PF-03084014 was well tolerated in mice at dose levels below 100 mg/kg. HPB-ALL xenotransplanted mice were tested at 150 mg/kg with different intermittent treatment schedules: continuous treatment, 3 days treatment/4 days withdrawal, 7 days treatment/7 days withdrawal. The PF-03084014 at 7-day treatment/7-day withdrawal schedule exerted the best anti-cancer efficacy and the least toxicity in HPB-ALL xenotransplanted mice [19]. In a phase I study, eight patients with T-ALL or T-cell lymphoblastic lymphoma (5 T-cell lymphoblastic lymphoma and 3 T-ALL) received PF-03084014 at 150 mg twice daily [20]. All eight patients had previously received systemic treatment and relapsed. One of the T-ALL patients achieved complete remission lasting about 3 months, and then relapsed. The *HES4* gene expression level was suppressed during remission and was higher than the baseline level when the disease relapsed in this case. The most common adverse effects were nausea and vomiting [20].

BMS-906024, another small molecule γ-secretase inhibitor was screened by efficacy/tolerance profile. The efficacy of BMS-906024 was dose-dependent, and the lowest effective dose was 1 mg/kg/day in xenotransplantation mice [21]. At higher doses, a significant delay in tumor growth can be observed even after the administration was complete. And no significant toxicity such as weight loss of mice was observed [21]. A clinical trial of the agent allowed a 53-year-old male patient with relapsed and refractory early T-cell progenitor acute lymphoblastic leukemia to reach complete remission with a deep molecular response [22]. In a Phase I clinical trial, BMS-906024 was administered in 25 children with T-ALL or T-cell lymphoblastic lymphoma at 4–6 mg weekly. One-third of cases (32%) showed at least a 50% reduction in bone marrow blasts and had tolerable side effects [23].

MRK-560, a precise selective γ-secretase inhibitor, mainly targets PSEN1 in the γ-secretase complex in T-ALL cell lines [24]. MRK-560 was used to treat T-ALL patient-derived xenograft mouse models with different *NOTCH1* mutations. The anti-tumor activity and improved survival were observed in all mice with various response. And the mice administered with MRK-560 did not show obvious gastrointestinal pathological changes and thymus T cell development defects [24].

There are currently a variety of GSIs in the experimental stage [5]. But overall, GSI has a limited anti-tumor effect, mostly manifested as transient growth arrest rather than cell death, and a single agent seems to be insufficient to eradicate T-ALL blasts [25]. Combining GSI with other agents that can trigger cell death may be an alternative option to treatment of *NOTCH1*-mutant T-ALL [26, 27]. Chloroquine (CQ) can increase the accumulation of reactive oxygen species in T-ALL, activate DNA damage, enhance GSI-induced cell cycle arrest in T-ALL, and interfere with ligand-independent NOTCH1 transportation and localization [28]. In addition, it can also reduce the concentration of GSI with less side effects [28]. The combination therapy of GSIs and CQ showed excellent synergistic effect in vitro on T-ALL cell line [28]. Because there is PI3K/mTOR pathway activation in T-ALL [29], some researchers have conducted experiments using GSI and mTOR inhibitor rapamycin in mouse model [30]. T-ALL xenograft mice were divided into 4 treatment groups: control group, GSI group, rapamycin group and combination group. The average survival time of the combination group was significantly longer than that of the single agent group and the control group. The combined treatment increased the mean survival time of mice by more than 50 days [30]. These results suggest that the combination of GSI and mTOR inhibitors have synergistic inhibitory effect on T-ALL blasts. The addition of glucocorticoid to GSI can overcome the severe gastric-intestinal toxicity of GSI in a xenograft mice model [19]. The xenograft mice were treated with PF-03084014 (150 mg/kg, twice a day) or PF-03084014 plus dexamethasone (15 mg/kg, once a day) [19]. After euthanizing of mice at day 14, the duodenum in combined treatment mice had similar number of goblet cells to that in untreated animals assayed by histology, which demonstrated that dexamethasone had a protective effect on gastrointestinal toxicity caused by PF-03084014 [19]. Furthermore, the application of GSI can reverse glucocorticoid resistance of T-ALL [31]. In the highly resistant human T-ALL cell line with glucocorticoids, when treated with dexamethasone concentrations as high as 1 × 10^–5^ M, cells only showed the minimum loss of cell viability. However, the addition of GSI to dexamethasone showed a synergistic dose-dependent response and could effectively reduce cell viability, with a 50% inhibitory concentration value of 7.7 × 10^–8^ M for dexamethasone in the presence of GSI. The addition of glucocorticoids antagonists can reverse the synergistic effect of combination therapy [32]. There are also other combination options tested. For example, protein kinase CK2 inhibitor CX-4945 combined with GSI can destroy the stability of NOTCH1, reduce the growth and survival of human T-ALL cells [33, 34]. The combined application of cyclin D-dependent kinase CD4 inhibitor and GSI made ALL cells more sensitive to GSI, with a synergistic effects on cell cycle arrest and cell death [35, 36]. Therefore, it is promising to apply GSI with other agents combining with chemotherapy in the management of T-ALL cases.

Currently, no GSI has been approved for the clinical use in treatment of T-ALL. In addition to gastrointestinal toxicity and limited anti-leukemia effects, researchers also found that there are differences in the degree of inhibition of NOTCH1 cleavage by various GSIs and a lack of bioequivalence [37]. Further study found that some T-ALL cells are resistant to GSIs, which may be related to PTEN inactivating mutations [26, 38]. Therefore, further researches on GSIs with different agents are necessary to guarantee the effective management of T-ALL cases with *NOTCH1* mutation.

### ADAM inhibitors

Notch receptor activation requires ADAM protease (ADAM10 or ADAM17) cleavage at the S2 site. Inhibition of the ADAM protease may block the Notch receptor activation. Therefore, G1254023X, an ADAM10 inhibitor has been investigated in T-ALL. The proliferative rate of Jurkat cells decreased significantly in a dose-dependent manner after treated with 20 μmoL/L G1254023X for 48 h. At the same time, 20 µmoL/L G1254023X has a stronger apoptosis-inducing effect than 10 µmoL/L G1254023X. Significantly higher level of intracellular NOTCH1 protein and lower level of cleaved NOTCH1 protein was observed in the treated cells as compared to the untreated cells. The transcription of *Hes-1*, one of the target genes of *NOTCH1*, was reduced by 80–90%. The results suggest that G1254023X induce apoptosis and inhibit proliferation by inhibiting the activation of NOTCH1 signaling pathway in Jurkat cells [39]. This data provide evidences for further exploration of ADAM10 inhibitor in T-ALL with *NOTCH1* mutation.

### Monoclonal antibody targeting NOTCH1

OMP-52M51 is a monoclonal antibody produced by mice immunized by human NOTCH1 protein fragments [40]. It can bind to the negative regulatory region of NOTCH1 and prevent ligand-free activation of NOTCH1 receptors. OMP-52M51 reduces the level of NOTCH1 intracellular domain in T-ALL cell line in vitro and significantly inhibits T-ALL cell growth in xenograft models [40]. Agnusdai and colleagues [40] reported seven T-ALL xenograft mice (4 *NOTCH1* mutants and 3 *NOTCH1* wild types) treated with OMP-52M51. All of the T-ALL xenograft mice carrying *NOTCH1*-mutant had reduced circulating leukemia cells, while the animals carrying *NOTCH1* wild-type had no response to OMP-52M51 treatment. The survival time of *NOTCH1*-mutant T-ALL mice were significantly prolonged in comparison to the animals carrying *NOTCH1* wild-type after treatment with OMP-52M51(mean 44 days vs. 15 days). Leukemia burden were reduced by 90% in animals with *NOTCH1*-mutant by administration of OMP-52M51. At necropsy, the spleen size was significantly reduced in mice treated with anti-NOTCH1 antibody compared to that in the untreated group. Ki67 staining showed a significant reduction after anti-NOTCH1 antibody administration. Similar to GSIs, the combined application of OMP-52M51 and dexamethasone enhances the anti-NOTCH1 efficacy [40]. However, some T-ALL xenografts developed resistance to OMP-52M51. And gene sequencing analysis suggested that OMP-52M51 resistant mice mostly carried two activation mutations of *NOTCH1* gene: p.Q1584H and p.L1585P [41]. Therefore, T-ALL patients with p.Q1584H and p.L1585P mutations of *NOTCH1* gene may not benefit from OMP-52M51. However, there are no further clinical trials to support this hypothesis, and further research is needed.

Ankur and colleagues generated a monoclonal antibody mAb604.107 against the negative regulatory region of NOTCH1 [42]. At low concentrations (1–2 μg/mL), it can distinguish the conformation of the NRR region between mutated *NOTCH1* and wild-type *NOTCH1*. T-ALL leukemia-initiating cells were inhibited by mAb604.107. The mAb604.107 can increase the sensitivity of the chemotherapy drug doxorubicin in an animal model [42]. Therefore, mAb604.107 may be a promising monoclonal antibody targeting *NOTCH1*-mutant in T-ALL.

### Direct inhibition of NOTCH1 transcription factor complex

ICN1 combines with CSL to form a long and shallow groove in cell nucleus, which serves as the binding surface of MAML. A synthetic cell-permeable α-helix peptide, SAHM1, targets the ternary complex of ICN1-CSL-MAML in NOTCH signaling pathway [43]. The α-helical peptide SAHM1 competes with MAML1 to bind to the ICN1-CSL complex, and blocks the formation of ICN1-CSL-MAML ternary complex, thus leading to the inhibition of *NOTCH1* target gene expression. In the T-ALL mouse model, cell proliferation was significantly reduced after SAHM1 treatment, while mRNA levels of *Heyl*, *Hes1*, *Myc*, *Dtx1* and *Nrarp* were significantly reduced [43]. And the animals treated with SAHM1 showed that the weight of the spleen and the absolute number of donor-derived circulating lymphoblasts were significantly reduced. Histopathological examination of bone marrow and spleen showed a significant reduction in leukemia infiltration. The T-ALL bioluminescent mouse model was treated with intraperitoneal injection of SAHM1 or placebo, once daily (35 mg/kg) or twice daily (30 mg/kg). The control mice experienced progressive disease, and 8/9 of them showed higher bioluminescence within 5 days. The mice treated with SAHM1 had less bioluminescence and fewer animals with progressive disease (4/6). These data suggest that targets in the complex of NOTCH signaling pathway may be an alternative option in management of T-ALL with over activation of NOTCH signals.

### SERCA inhibitors

The formation of the NOTCH1 receptor protein HD region and the transport process of NOTCH1 require the participation of calcium ions [17]. The NOTCH1 signal is related to the intracellular Ca^2+^ homeostasis in T-ALL [44]. In a NOTCH1-dependent model of T-ALL, the absence of Ca^2+^ channel activators STIM1 and STIM2 can significantly prolong the survival of these animals [45]. Ca^2+^ ATPase (SERCA) regulates the calcium ion concentration. Therefore, some studies explore the role of SERCA inhibitors in activation of NOTCH1 signaling pathway.

The compound thapsigargicin was screened from a series of SERCA inhibitors. The study in vitro showed that thapsigargicin induced cellular apoptosis by depletion of endoplasmic reticulum calcium ions and oxidative stress. The inhibitory effect of thapsigargicin on T-ALL was evaluated in cell lines and mouse model [46]. Thapsigargicin inhibits *NOTCH1* mutated T-ALL cells more specifically than *NOTCH1* wild-type T-ALL cells. However, severe dose-limited cardiotoxicity was observed, which limited the clinical application [47]. The folate conjugate named as JQ-FT combines folic acid with thapsigargicin through a cleavable bond to achieve leukemia-specific delivery of thapsigargicin. JQ-FT is a *NOTCH1* inhibitor with a dual selectivity, targeting both *NOTCH1* mutations and leukemia cells. In the xenograft model, JQ-FT inhibits *NOTCH1*-mutated T-ALL growth in vivo. And the sensitivity of JQ-FT to cells in *NOTCH1*-mutated T-ALL is higher than that of *NOTCH1* wild-type cells [47].

Clerodane diterpene casearin J (CJ), a natural SERCA inhibitor, mainly targets *NOTCH1* HD domain mutant in T-ALL. Experiments in cell lines show that CJ can induce T-ALL cell death in the low molar concentration [48]. CJ reduces the level of ICN1 in T-ALL cells carrying *NOTCH1* HD domain mutations, and this effect was significantly reduced in the cells with normal *NOTCH* alleles and juxtamembrane expansion mutations [49]. CJ can also cause slight activation of *NF-κB*. The combined treatment of CJ and NF-κB inhibitor, parthenolide, resulted in significant synergistic death of T-ALL cells [48].

Recently, Marchesini and colleagues identified an oral SERCA inhibitor, CAD204520 [50]. The toxicity of CAD204520 to off-target calcium ions is significantly reduced. T-ALL xenograft mice treated with CAD204520 by oral gavage (dose 45 mg/kg, twice a day, 8 h apart, for 4 days) showed that the percentages of circulating leukemia cells were 56-fold reduced compared to the control group. Leukemia infiltration in the spleen also decreased significantly. There were no weight loss, no adverse effects on behavior, and no signs of acute cardiotoxicity or gastrointestinal metaplasia observed in mice treated with CAD204520. The complete blood count of the treatment group had no significant difference from control group. There were no general pathological abnormalities in internal organs, including heart, lung, liver, brain and kidney at 21 days after the treatment. These data demonstrated that CAD204520 is a promising option in the management of T-ALL with NOTCH mutation due to its high efficacy and tolerable toxicities.

### Other agents

In addition to the above studies, there are other experimental protocols targeting *NOTCH1* [51, 52], such as the proteasome inhibitor (bortezomib) [53], histone deacetylase inhibitor (panobinostat) [54], HSP90 inhibitor [55, 56], insecticide (mebendazole) [57], geranylgeranyl diphosphate synthase inhibition (digeranyl bisphosphonate, DGBP) [58], and the antibody Rova-T against its ligand DLL3 ^[^59^]^. Other natural anti-*NOTCH* compounds have been shown to inhibit *NOTCH1* mutant T-ALL cells, such as plant polyphenol flavonoids [60], artemisinin [61], etc. However, all of the currently investigated agents targeting on Notch signaling pathway are waiting to be approved for clinical application in the management of T-ALL patients [2].

## Conclusion and prospect

*NOTCH1* gene plays an important role in lymphocyte differentiation, development and proliferation [62, 63]. Mutations in *NOTCH1* gene play a key role in the occurrence and progression of ALL, especially T-ALL [64, 65]. Therefore, targeting the *NOTCH1* signaling pathway in T-ALL has been a research focus in recent years. The complex mechanism of *NOTCH1* and its signaling pathway in T-ALL also provides many treatment options for this disease (Table 1). Researchers have developed GSIs, ADAM10 inhibitors, monoclonal antibodies, SERCA inhibitors, a-helical peptides that inhibit NOTCH1 transcription factor complex, and other agents to interfere with the NOTCH signaling pathway. Although the clinical trials of GSIs alone have shown limited anti-tumor efficacy and dose-limiting toxicity, the development in GSIs and the combination with other agents showed some promising therapeutic effects. Meanwhile, other agents in preclinical trials targeting NOTCH1 signaling pathway have shown efficacy against T-ALL. Since each agents has its own limitations (Table 2), none of the agents targeting NOTCH1 signaling pathway in T-ALL is currently applied in clinical practice. But it is undeniable that targeted therapy on NOTCH1 signaling pathway is showing promises for a breakthrough in T-ALL management. It is expected that further investigation in the field will significantly benefit the T-ALL patients.

**Table 1:** Agents targeting *NOTCH1* pathway in T-ALL

Name	Target	Mechanism	Type of cancer	Phase	NCT number/publication date	Assessment
MK-0752	γ-Secretase	Induce G0/G1 cell cycle arrest and inhibit cell proliferation	T-ALL	I	2006	Transient anti-leukemia effect but severe diarrhea
PF-03084014	γ-Secretase	Inhibit ICN1 levels and the expression of Notch target genes	T-ALL or T-cell lymphoblastic lymphoma	I	NCT00878189	1/8 achieved complete remission lasting about 3 months and the most common adverse effects were nausea and vomiting
BMS-906024	γ-Secretase	γ-Secretase inhibitor screened by efficacy/tolerance profile	T-ALL or T-cell lymphoblastic lymphoma	I	NCT01363817	32% showed at least a 50% reduction in bone marrow blasts and had tolerable side effects
MRK-560	PSEN1 of γ-Secretase	Selective γ-Secretase inhibitor, mainly target PSEN1 in the γ-secretase complex	T-ALL	Preclinical	July 1, 2019	Improved survival and did not show obvious gastrointestinal pathological
G1254023X	ADAM	Prevent ADAM protease cleavage Notch receptor at the S2 site	T-ALL	Preclinical	August 20, 2015	Inhibited the activation of NOTCH1 signaling pathway and induce apoptosis
OMP-52M51	NRR	Prevent ligand-free activation of NOTCH1 receptors	T-ALL	Preclinical	July 23, 2013	Prolonged survival time but had drug resistance
MAb 604.107	NRR	Distinguish the conformation of the NRR region between mutant NOTCH1 and wild-type NOTCH1	T-ALL	Preclinical	June 5, 2015	Inhibited T-ALL leukemia-initiating cells
SAHM1	ICN1-CSL-MAML complex	Block the formation of ICN1-CSL-MAML complex, and inhibit the activation of NOTCH1 target gene expression	T-ALL	Preclinical	November 12, 2009	Inhibited leukemic progression and NOTCH1 signaling
Thapsigargicin	SERCA	Induce the depletion of endoplasmic reticulum calcium ions and oxidative stress, which ultimately leads to apoptosis	T-ALL	Preclinical	March 18, 2013	Effective but severe cardiotoxicity
JQ-FT	SERCA	Combine folic acid and thapsigargicin with a cleavable bond to achieve leukemia-specific delivery of thapsigargicin	T-ALL	Preclinical	January 2, 2018	Dual selectivity: targeting *NOTCH1* mutations and targeting leukemia cells, but Complex process and poor practicality
CJ	SERCA	Target NOTCH1 HD domain mutant T-ALL, induce T-ALL cell death	T-ALL	Preclinical	January 28, 2016	Mainly target NOTCH1 HD domain mutation but weak effect on cells with mutations in other domains of NOTCH1
CAD204520	SERCA	Retain the anti-NOTCH1 tumor characteristics while inhibiting thapsigargicin-resistant cell lines	T-ALL	Preclinical	June 18, 2020	Effective and had tolerable side effects

**Table 2:** Adverse events for each agent

Type of agents	Agents	Adverse events
γ-Secretase inhibitors (GSIs)	MK-0752	Gastrointestinal toxicity (mainly diarrhea)
	PF-03084014	Nausea and vomiting
	BMS-906024	Tolerable gastrointestinal toxicity
	MRK-560	Did not show obvious gastrointestinal pathological changes and thymus T cell development defects
ADAM inhibitors	G1254023X	No records of adverse events related to the drug found in the literature
Monoclonal antibody targeting NOTCH1	OMP-52M51	May be resistant to T-ALL of carried two activation mutations of NOTCH1 gene: p.Q1584H and p.L1585P
	mAb604.107	No records of adverse events related to the drug found in the literature
Direct inhibition of NOTCH1 transcription factor complex	SAHM1	No records of adverse events related to the drug found in the literature
SERCA inhibitors	Thapsigargicin	Severe dose-limited cardiotoxicit
	JQ-FT	Complex process and poor practicality
	Clerodane diterpene casearin J (CJ)	No records of adverse events related to the drug found in the literature
	CAD204520	No weight loss, no adverse effects on behavior, and no signs of acute cardiotoxicity or gastrointestinal metaplasia

## Data Availability

The material supporting the conclusion of this review has been included within the article.

## Abbreviations

T-ALL: T-cell acute lymphoblastic leukemia
CAR-T: Chimeric antigen receptor modified T
EGF: Epidermal growth factor
NRR: Negative regulatory region
LNR: Lin12-Notch repeats
HD: Heterodimerization domain
PEST domain: Proline/glutamic acid/serine/threonine enriched motif
ADAM: A disintergrin and metalloprotease
GSI: γ-Secretase inhibitor
ICN1: Intracellular domain of NOTCH1
CQ: Chloroquine
CSL: C-promoter binding factor 1 CBF-1, suppressor of hairless, lag
MAML: Mastermind-like
SERCA: Ca^2^^+^ ATPase
CJ: Clerodane diterpene casearin J
